# Laser Acupuncture on the Management of Xerostomia in Sjögren's Disease: A Randomized Clinical Trial

**DOI:** 10.1111/odi.70241

**Published:** 2026-04-20

**Authors:** Matheus Ferreira Linares, Tainá Duarte dos Santos França, Maria Clara Barros Madureira Ferreira, Michele Gomes do Nascimento, Angela Luzia Branco Pinto Duarte, Luiz Alcino Gueiros

**Affiliations:** ^1^ Department of Oral Diagnosis, Semiology and Pathology Areas, Piracicaba Dental School University of Campinas (UNICAMP) Piracicaba São Paulo Brazil; ^2^ Private Office Recife Pernambuco Brazil; ^3^ Department of Pediatric Dentistry University of Pernambuco Recife Pernambuco Brazil; ^4^ Rheumatology Unit, Hospital das Clínicas Universidade Federal de Pernambuco Recife Pernambuco Brazil; ^5^ Department of Clinic and Preventive Dentistry, Oral Medicine Unit Federal University of Pernambuco (UFPE), School of Dentistry Recife Pernambuco Brazil

**Keywords:** acupuncture, phtotobiomodulation, Sjögren's syndrome, xerostomia

## Abstract

**Objectives:**

This study evaluated the efficacy of photobiomodulation (PBM) at acupuncture points for managing xerostomia in patients with Sjögren's Disease.

**Material and Methods:**

In this randomized, double‐blind clinical trial, 50 patients were assigned to PBM group (*n* = 22) or sham‐PBM group (*n* = 28). The primary outcome was xerostomia symptoms assessed using a visual analogue scale (VAS). Secondary outcomes included Xerostomia Inventory, resting salivary flow rate (RSFR), and EULAR Sjögren's Syndrome Patient Reported Index score. Participants received weekly PBM sessions for four consecutive weeks, and evaluations were conducted at baseline, week 8 and 12.

**Results:**

PBM showed a marginal tendency toward improvement on VAS (*p* = 0.0974) (OR = 1.929 95% CI 0.8939–4.525) and in the RSFR (*p* = 0.09996) (OR = +infinity 95% CI 0.9363‐infinity) at week 8 compared with sham‐PBM. However, no statistically significant differences were observed between groups at week 12 for any of the assessed outcomes.

**Conclusion:**

PBM at acupuncture points demonstrated a slight short‐term benefit at week 8 in improving xerostomia symptoms and RSFR, although these effects did not persist at 12 weeks. These preliminary results suggest a potential role for PBM at acupuncture points as a safe, noninvasive adjunctive therapy for xerostomia in patients with SjD, warranting further validation in larger, long‐term trials.

**Trial Registration:**

This study was registered in The Brazilian Registry of Clinical Trials (U1111‐1298‐9891)

## 1 Introduction

With a prevalence ranging from 0.1% to 4.8%, Sjögren's Disease (SjD) is one of the most common rheumatic disorders (Mavragani and Moutsopoulos 2014; Ramos‐Casals et al. 2012). This autoimmune condition primarily affects middle‐aged women and is characterized by lymphocytic infiltration of the salivary and lacrimal glands, resulting in decreased saliva and tear production (Wei et al. 2018). As a result, patients experience distressing symptoms of dry eyes (xerophthalmia) and dry mouth (xerostomia), collectively known as sicca syndrome (Baer and Walitt 2018; Baldini et al. 2024; Brito‐Zerón et al. 2016; Mavragani and Moutsopoulos 2014; Thorlacius et al. 2023). The non‐specific nature of SjD symptoms, often resembling those of other conditions, frequently leads to delayed diagnosis (Petruzzi et al. 2023). Therefore, accurate diagnosis relies on a combination of clinical and laboratory criteria, including labial salivary gland biopsy, Anti‐Ro/SSA, ocular staining, Schrimer's and resting salivary flow rate (Baer and Walitt 2018; Brito‐Zerón et al. 2016; Mavragani and Moutsopoulos 2014; Thorlacius et al. 2023). Given that extra‐glandular manifestations occur in approximately 50% of cases (Baer and Walitt 2018), treatment primarily focuses on symptomatic relief, management of complications associated with reduced saliva and tear production, and stimulating residual gland function. However, medications used for these purposes, such as pilocarpine and cevimeline, are frequently associated with adverse events like sweating and nausea and often provide limited clinical benefit, particularly in advanced stages of glandular dysfunction (Baer and Walitt 2018; Baldini et al. 2024; Brito‐Zerón et al. 2016; Mavragani and Moutsopoulos 2014; Thorlacius et al. 2023).

Given the limitations of conventional treatments for SjD, alternative approaches, such as Traditional Chinese Medicine (TCM), one of the oldest healing systems in the world, have been explored (Ma et al. 2016; Tang et al. 2008). TCM, treatment modalities include acupuncture, massage, dietary therapy, and physical exercise (Ma et al. 2016; Tang et al. 2008). Studies have demonstrated that acupuncture exerts physiological, anatomical, and neurochemical effects, supporting its application as a medical approach (Zhao 2008). In a systematic review, Assy and Brand (2018) reported that studies evaluating acupuncture for xerostomia/hyposalivation exhibited a high risk of bias. Despite these limitations, current evidence still supports the potential of acupuncture to increase salivary flow, particularly in patients undergoing radiotherapy. Furthermore, the most recent guidelines for managing xerostomia in cancer patients have indicated that acupuncture may be an effective treatment option for this cancer population (Mercadante et al. 2021). While evidence for acupuncture in SjD‐related xerostomia is scarce, a sham‐controlled RCT has demonstrated its efficacy for dryness relief after 4 weeks of completing the treatment (Gomes‐Silva et al. 2025). Nevertheless, it has been suggested that future well‐designed studies are needed to better establish the therapeutic potential of acupuncture for hyposalivation (Assy and Brand 2018).

Additionally, photobiomodulation (PBM) is a therapeutic approach that uses nonthermal red or infrared light to stimulate or suppress biological processes, primarily through mitochondrial activation, enhanced ATP production, modulation of inflammatory pathways, and improved microcirculation, leading to cellular repair and functional restoration (Pandeshwar et al. 2016; Zecha et al. 2016a, 2016b). In the field of oral medicine, PBM has been investigated as a treatment option for various conditions, including oral mucositis, herpes simplex, oral lichen planus, recurrent aphthous stomatitis, and hyposalivation (Kalhori et al. 2019). Studies have demonstrated positive outcomes of PBM as an intervention for xerostomia/hyposalivation, particularly when these symptoms are associated with medication use, diabetes, and head and neck cancer treatment. However, only a limited number of studies have explored its efficacy on SjD patients (Biswas et al. 2018; Dabić et al. 2016; Lončar et al. 2011; Palma et al. 2017; Sousa et al. 2020). Nevertheless, combining PBM and acupuncture is not a novelty due to the ability of laser irradiation to induce cellular effects at subthermal thresholds, and has been used in several clinical conditions, including osteoarthritis, Bell's palsy, and SjD, with promising results (Cafaro et al. 2015; Wu et al. 2024, 2020). In this context, there appears to be a synergistic rationale for combining acupuncture and PBM to potentially enhance their therapeutic benefits. Given these considerations, the present study aimed to conduct a randomized clinical trial to evaluate the efficacy of PBM applied to acupuncture points for managing xerostomia of patients diagnosed with SjD, thereby contributing a new alternative to the clinical arsenal.

## 2 Materials and Methods

### 2.1 Study Design

This was a single‐center, randomized, double‐blind clinical trial conducted at the Rheumatology Unit of Hospital das Clínicas—Universidade Federal de Pernambuco (UFPE), Recife, Brazil, between October 2023 and December 2024. The study was registered in the Brazilian Registry of Clinical Trials and received ethical approval from the Research Ethics Committee of Hospital das Clínicas—UFPE (7.126.862). The study adhered to the principles of the Declaration of Helsinki, and written informed consent was obtained from all participants.

### 2.2 Study Participants

Participants were recruited from the SjD outpatient clinic at the Rheumatology Unit of Hospital das Clínicas (UFPE). Inclusion criteria comprised patients diagnosed with SjD according to the 2016 American College of Rheumatology/European League Against Rheumatism (ACR/EULAR) classification criteria (Shiboski et al. 2017) who were 18 years or older. Exclusion criteria included current pharmacological treatment for SjD symptoms, coexistence of another autoimmune rheumatic disease, previous head and neck radiation therapy, hepatitis B or hepatitis C infection, HIV infection, and sarcoidosis. As this was a preliminary study, no formal sample size calculation was performed, and a convenience sample was used.

### 2.3 Randomization and Blinding

Simple randomization with equal allocation was employed. Participants were randomly assigned to either the placebo or intervention group. Randomization was performed using a random computer‐generated list from 1 to 60, created at www.random.org. Odd numbers were assigned to the laser group, and even numbers to the sham‐laser group. Following the sequence of the randomization list, each participant received an opaque, sealed, and numbered envelope containing their assigned treatment group after signing the informed consent form. Both the participant and the outcome assessor (ML) were blinded to the treatment. The professional administering PBM was the only member of the clinical team aware of the allocation. All outcomes were recorded by an independent examiner who was also blinded to the intervention.

### 2.4 Intervention

#### 2.4.1 Pilot Study

**FIGURE 1:**
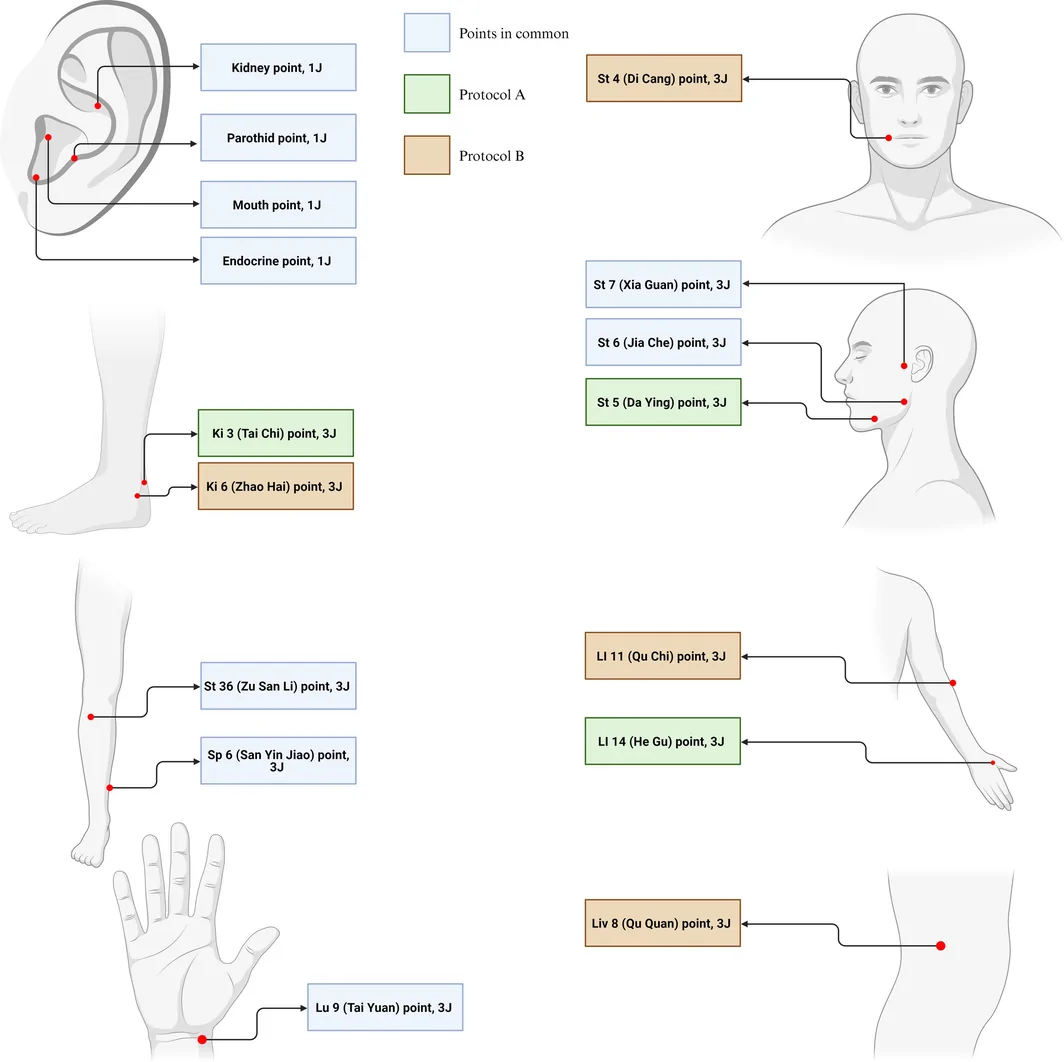
Protocol/acupoints used. Laser and sham laser groups had the same points stimulation, though sham laser had no light emission. Protocol B was selected given its marginal tendency of significance (*p* = 0.088), as Protocol A presented a *p* value of 0.693. Intervention duration: 1 session per week, for 4 weeks. Laser parameters used (Both protocols): Gallium Aluminum Arsenide (GaAlAs) laser; wave length: 808 nm (infrared); output power: 100 mW; laser area: 0.03 cm^2^; Irradiation time: 30 s for extra‐auricular points (3 J/point) and 10 s for auricular points (1 J/point).

Prior to the RCT, a pilot study was conducted with healthy volunteers to determine the most effective acupoints for improving resting salivary flow rate (RSFR). This was a single‐center, cross‐sectional study. Participants had their RSFR measured before the treatment and immediately after treatment with laser acupuncture. The intervention was performed using a Gallium Aluminum Arsenide (GaAlAs) laser, with a wavelength of 808 nm (infrared), an output power of 100 mW, a laser spot area of 0.03cm^2^, and an irradiation time of 30 s for extra‐auricular points (3 J/point) and 10 s for auricular points (1 J/point), totalling 60 J per session. The specific protocol and acupoints used for each group are detailed in Figure 1. Data from the pilot study are summarized in Table 1.

**TABLE 1:** Pilot study data.

Sex	Age (years)	Protocol	Pre‐treatment RSFR	Post‐treatment RSFR
F	54	A	0.1 mL	0.2 mL
M	52	A	0.6 mL	1.0 mL
F	23	B	0.9 mL	0.6 mL
F	49	B	0.1 mL	0.2 mL
M	62	A	0.2 mL	2.1 mL
F	34	A	0.1 mL	0.2 mL
F	63	B	0.1 mL	0.4 mL
M	61	B	0.2 mL	1.3 mL
F	68	B	0.2 mL	0.2 mL
M	50	B	1.5 mL	1.0 mL
F	56	B	1.0 mL	3.1 mL
F	34	A	1.0 mL	0.8 mL
F	48	A	0.6 mL	0.5 mL
F	47	A	0.6 mL	1.0 mL
F	32	B	0.7 mL	1.6 mL
F	41	A	0.5 mL	0.3 mL
M	56	B	0.5 mL	0.5 mL
F	40	A	0.1 mL	0.2 mL
M	39	B	1.0 mL	2.2 mL
F	66	A	1.2 mL	1.8 mL

Abbreviation: RSFR, resting salivary flow rate.

The results from each group were analyzed using the paired Wilcoxon test to compare pre‐ and post‐test results. Protocol A had a *p* value of 0.693 (RSFR median pretreatment = 0.55 mL, and RSFR median posttreatment = 0.65 mL), whereas protocol B had a *p* value of 0.088 (RSFR median pretreatment = 0.60 mL, and RSFR median posttreatment = 0.80 mL). Even though no statistically significant value was seen in any group, protocol B was selected given its marginal trend toward significance.

#### 2.4.2 Clinical Trial

For the RCT, PBM was performed using a gallium aluminum arsenide laser (GaAlAs, DMC Photon Lase III) emitting infrared light at 808 nm, with an output power of 100 mW, and a beam area of 0.03cm^2^. The irradiance was 3.33 W/cm^2^, with an irradiation time of 30 s for extra‐auricular points and 10 s for auricular points. The fluence was 100 J/cm^2^ for extra‐auricular points and 33.33 J/cm^2^ for auricular points. A total energy of 3 J per extra‐auricular point and 1 J per auricular point was applied, totalling 60 J per session and a cumulative dose of 240 J per treatment. The acupuncture protocol, illustrated in Figure 1, was developed by a dentist specialized in TCM. Each point was irradiated bilaterally. Patients allocated to the placebo group received the same acupuncture protocol; however, no light was emitted from the laser device. Blinding effectiveness was assessed at the end of the trial by directly questioning both patients and the investigator.

### 2.5 Outcomes

#### 2.5.1 Primary Outcome

The primary outcome was a subjective assessment of dry mouth symptoms using a Visual Analogue Scale, with a clinically significant improvement defined as a change greater than 14 mm (Mercadante et al. 2016).

#### 2.5.2 Secondary Outcomes

Secondary outcomes included objective measures such as the Xerostomia Inventory (XI), for which a difference of ≥ 6 points was considered clinically significant (Thomson 2007); the RSFR with an improvement of salivary flow > 0.1 mL/min regarded as clinically significant (Dawes 1987; Mercadante et al. 2021); and the total score of the EULAR Sjögren's Syndrome Patient Reported Index (ESSPRI), for which a difference of ≥ 1 was considered clinically significant (Seror et al. 2011, 2015). Outcome values were collected at baseline and post‐treatment (weeks 8 and 12). The VAS, XI, and ESSPRI are validated xerostomia‐related, patient‐reported outcome measures that assess the impact of these symptoms on patient quality of life (Amaral et al. 2018; da Mata et al. 2012; Mercadante et al. 2016; Seror et al. 2011; Thomson 2007).

### 2.6 Statistical Analysis

Statistical analyses were performed using Graphpad Prism software (version 10.4.2). All analyses were carried out on a per‐protocol basis. Proportions were compared using Fisher's exact test and odds ratio with corresponding 95% confidence intervals. Given the sample size, non‐parametric tests were applied. The Mann–Whitney test was used to compare the median value between two groups, and the Kruskal‐Wallis test was used to compare medians among 3 groups. The number needed to treat (NNT) was calculated using the formula: NNT = 1/ARR (Absolute Risk Reduction). The null hypothesis was rejected at a significance level of *p* < 0.05.

## 3 Results

### 3.1 CONSORT Flowchart and Baseline Comparison

**FIGURE 2:**
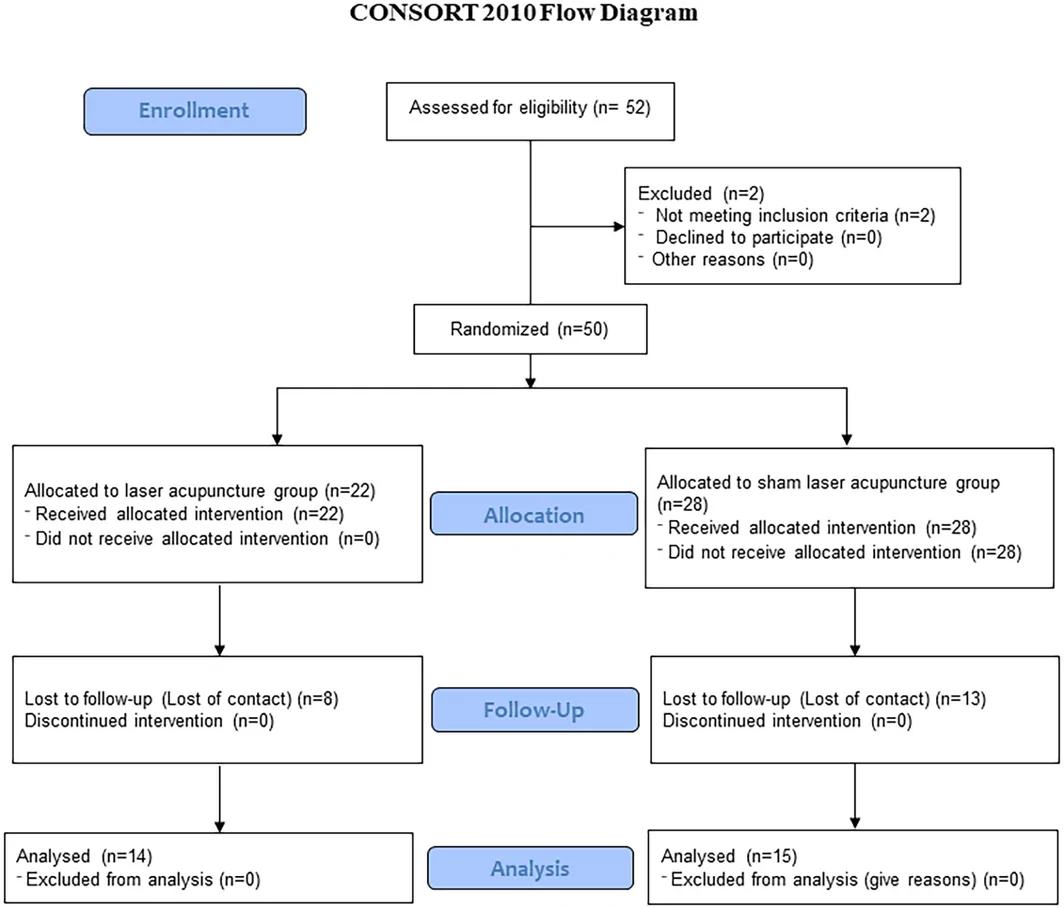
Study flowchart.

This single‐center, two‐arm, double‐blind clinical trial was conducted between October 2023 and December 2024. As shown in Figure 2, a total of 52 patients were initially recruited. Two patients were excluded for not meeting the diagnostic criteria, resulting in 50 eligible participants. These participants were then randomized to receive either laser acupuncture (*n* = 22) or sham laser acupuncture (*n* = 28). At week 4, eight participants withdrew from the laser acupuncture group, and thirteen from the sham laser acupuncture group, all due to loss of contact. At week 12, one participant from each group withdrew, all due to loss of contact. The baseline epidemiologic characteristics are presented in Table 2.

**TABLE 2:** Epidemiologic characteristics.

	Laser acupuncture	Sham laser acupuncture	*p*
Number of patients randomized	22	28	
Age, mean (SD), years	53.14 (8.5)	50.46 (10.7)	0.6812
Age, range, years	36–76	26–65	
Female, *n* (%)	22 (100)	28 (100)	> 0.9999
Alcohol consumption, yes (%)	0 (0)	2 (7.14)	0.4971
Tobacco consumption, yes (%)	1 (4.54)	1 (3.57)	> 0.9999
Xerostomia, yes (%)	22 (100)	27 (96.43)	> 0.9999
Xerophthalmia, yes (%)	21 (95.45)	25 (89.28)	0.6209
Sialometry, mean (SD), mL	0.063 (0.075)	0.074 (0.105)	0.7566
Schirmer's test, mean (SD), mm	5.59 (9.11)	3.86 (7.75)	0.3617
Anti‐Ro antibodies, positive (%)	13 (59.09)	18 (64.28)	0.7742
Salivary gland biopsy, positive (%)	12 (54.54)	9 (32.14)	0.1520
Parotid enlargement, positive (%)	6 (27.27)	12 (42.86)	0.3744
Arthralgia, positive (%)	16 (72.73)	22 (78.57)	0.7432
Vaginal dryness, positive (%)	13 (59.09)	23 (82.14)	0.1127
Raynaud phenomenon, positive (%)	15 (68.19)	16 (57.14)	0.5595
Dyspnea, positive (%)	13 (59.09)	17 (60.71)	> 0.9999
Number of drugs used, mean (SD), *n*	6 (2.76)	5.86 (2.9)	0.9630
XI, mean (SD), score	41.95 (7.59)	43.36 (8.09)	0.5478
ESSPRI, mean (SD), score	8.5 (1.05)	8.46 (1.21)	0.9760

Abbreviations: ESSPRI, EULAR Sjögren's Syndrome Patient Reported Index; SD, standard deviation; XI, xerostomia inventory.

### 3.2 Outcome Assessment

Outcome data were analyzed per protocol and are summarized below.

#### 3.2.1 Primary Outcome

Regarding the primary outcome, at week 8, 64.29% of patients in the PBM acupuncture group achieved the primary outcome endpoint, compared with 33.33% in the sham‐PBM acupuncture group. These results demonstrated a marginal trend toward benefit for VAS score (*p* = 0.0974), with an NNT of 3.231 and an odds ratio (OR) of 1.929 (95% confidence interval (95% CI) 0.8939–4.525), as shown in Table 3. However, no improvement was observed at week 12. Interestingly, the variance test also showed a marginal trend toward benefit in the VAS score for the laser acupuncture group (Table 4).

**TABLE 3:** Proportion of patients achieving the assessed outcomes at different weeks.

Outcome	Laser acupuncture	Sham laser acupuncture	RR (95% CI)	AR (95% CI)	NNT	*p*
VAS						
Week 8	9/14	5/15	1.929 (0.8939–4.525)	0.3095 (−0.09099 to 0.6072)	3.231	0.0974
Week 12	6/14	7/15	0.9184 (0.4004–2.055)	0.03810 (−0.3154 to 0.4056)	26.25	0.5664
XI						
Week 8	2/14	3/15	0.7143 (0.1569–3.139)	0.05714 (−0.2524 to 0.3872)	17.50	0.5394
Week 12	4/14	4/15	1.071 (0.3435–3.328)	0.01905 (−0.3234 to 0.3627)	52.50	0.6167
RSFR						
Week 8	3/14	0/15	+infinity (0.9363‐infinity)	0.2143 (−0.08398 to 0.5119)	4.667	0.0996
Week 12	0/14	2/15	0.000 (0.000–1.876)	0.1333 (−0.1495 to 0.4226)	7.500	0.2586
ESSPRI						
Week 8	2/14	4/15	0.5357 (0.1262–2.139)	0.1238 (−0.1846 to 0.4687)	8.077	0.3605
Week 12	3/14	4/15	0.8036 (0.2288–2.736)	0.05238 (−0.2731 to 0.3989)	19.09	0.5424

Abbreviations: AR, attributable risk; CI, confidence interval; ESSPRI, EULAR Sjögren's Syndrome Patient Reported Index; RR, relative risk; RSFR, resting salivary flow rate; VAS, visual analogue scale; XI, xerostomia inventory.

**TABLE 4:** Analysis of variance test for outcomes by groups.

	Baseline	Week 8	Week 12	*p*
VAS				
Laser acupuncture				
Minimum	27 mm	0 mm	6 mm	0.0917
Maximum	100 mm	86 mm	98 mm	
Median	67 mm	50 mm	48 mm	
Sham laser acupuncture				
Minimum	15 mm	6 mm	10 mm	0.9469
Maximum	100 mm	100 mm	100 mm	
Median	69 mm	59 mm	47 mm	
XI				
Laser acupuncture				
Minimum	22	22	13	0.5312
Maximum	55	54	50	
Median	43.5	42	45	
Sham laser acupuncture				
Minimum	25	26	22	0.1378
Maximum	51	53	53	
Median	44	41	41	
RSFR				
Laser acupuncture				
Minimum	0.000 mL/min	0.008 mL/min	0.008 mL/min	0.9813
Maximum	0.295 mL/min	0.356 mL/min	0.260 mL/min	
Median	0.050 mL/min	0.036 mL/min	0.345 mL/min	
Sham laser acupuncture				
Minimum	0.000 mL/min	0.000 mL/min	0.004 mL/min	0.8071
Maximum	2.870 mL/min	2.477 mL/min	0.702 mL/min	
Median	0.063 mL/min	0.063 mL/min	0.054 mL/min	
ESSPRI				
Laser Acupuncture				
Minimum	6.33	6.00	5.00	0.8353
Maximum	9.33	10.0	9.67	
Median	8.50	8.16	8.50	
Sham laser acupuncture				
Minimum	5.00	2.00	2.33	0.8669
Maximum	10.0	10.0	10.0	
Median	8.33	8.67	8.33	

Abbreviations: ESSPRI, EULAR Sjögren's Syndrome Patient Reported Index; RSFR, resting salivary flow rate; VAS, visual analogue scale; XI, xerostomia inventory.

#### 3.2.2 Secondary Outcomes

For the secondary endpoints, 21.43% of patients in the PBM acupuncture group and 00.00% of the sham‐PBM acupuncture group achieved the RSFR endpoint at week 8. These results sustained a marginal trend toward benefit (*p* = 0.0996) at week 8, with an NNT of 4.667 and OR = +infinity (95% CI 0.9363–infinity). Similar to the primary outcome, no sustained effect was detected at week 12.

For XI score 14.29% of patients in the PBM acupuncture group and 20.00% in the sham PBM acupuncture group achieved the endpoint at week 8, with no clear benefit (*p* = 0.5394). As with the previous outcomes, a decrease in statistical significance was observed at week 12 (*p* = 0.6167).

Lastly, for the ESSPRI score, 14.29% of participants in the PBM acupuncture group and 26.67% in the sham‐PBM acupuncture group reached the endpoint at week 8. No statistically significant differences were observed at week 8 (*p* = 0.3605) or week 12 (*p* = 0.5424). These results are summarized in Table 3.

## 4 Discussion

This study aimed to investigate the efficacy of PBM on patient‐perceived xerostomia in individuals with SjD and its subsequent impact on quality of life. It is among the few studies reported in the literature to address this issue. Additionally, it evaluated factors associated with disease activity. Overall, the present trial demonstrated that PBM had a marginal effect on patient quality of life and a potential benefit on VAS score and RSFR at week 8.

The management of xerostomia in SjD remains challenging due to the limited availability of effective treatment options (Plemons et al. 2014). The overall efficacy of pharmacological therapies has been modest, although some agents have shown promising results. Sialagogues such as pilocarpine have demonstrated some benefit in increasing salivary flow, but their effect on lacrimal gland function is limited and based on a small number of studies with methodological concerns (Wu and Li 2024). However, pilocarpine is associated with a high frequency of adverse events, which can preclude its use and limit its overall clinical impact (Choudhary et al. 2023). Among immunobiological therapies, rituximab has shown some benefit, while belimumab and abatacept have demonstrated potential efficacy in improving glandular function in patients with SjD (Gueiros et al. 2019). Novel immunobiologics, such as ianalumab (Bowman et al. 2022) and iscalimab (Fisher et al. 2024), have shown promising results and may become optimal therapeutic options in the near future. Nevertheless, these strategies are associated with high cost and potentially severe adverse events, underscoring the clinical need for alternative therapeutic approaches.

PBM has also been investigated as a treatment for xerostomia. In a systematic review, Sousa et al. (2020), reported that PBM may be effective in improving salivary flow and relieving xerostomia symptoms in SjD patients, being both safe and well‐tolerated (Sousa et al. 2020). However, other studies have not observed positive effects of PBM therapy in this population. Fidelix and colleagues (2018), reported no significant improvement on salivary gland function or salivary flow rate. The low baseline salivary flow rates observed in their study population were suggested as a possible explanation for this lack of benefit (Fidelix et al. 2018).

In this context, other non‐pharmacological therapies have been investigated for their effectiveness in alleviating dry mouth symptoms. In a Cochrane review, Furness et al. (2013) evaluated a range of interventions with variable results. The authors reported that acupuncture increased saliva production in patients experiencing xerostomia following radiotherapy (Furness et al. 2013). However, the review found insufficient evidence to determine the effects of electrostimulation on xerostomia or salivary production in patients with SjD. Although both acupuncture and electrostimulation showed some promising results, adverse events from these interventions were generally mild and transient (Furness et al. 2013). In a systematic review, Hackett et al. (2015) reported that neither intra‐oral lubricating devices nor psychodynamic group therapy improved the quality of life of patients diagnosed with SjD (Hackett et al. 2015).

Several studies have investigated the efficacy of acupuncture in managing xerostomia in SjD patients, yielding varying results. Given the holistic nature of TCM, which considers the interplay of multiple factors in health and disease, the optimal approach to managing SjD may vary considerably (Ma et al. 2016; Tang et al. 2008). This diversity in therapeutic strategies may explain the variability observed in acupuncture outcomes among patients with SjD, particularly regarding the selection of acupoints (Cafaro et al. 2015; Jiang et al. 2017; List et al. 1998; Liu et al. 2023; Zhou et al. 2022). Zhou et al. (2022) did not observe a satisfactory improvement in symptoms compared with placebo treatment (Zhou et al. 2022), whereas Liu et al. (2023) reported that TCM produced better clinical outcomes than Western medicine (Liu et al. 2023). Although traditional sham acupuncture is a viable method as a control procedure (Jiang et al. 2017), sham laser acupuncture was used in this study. This decision was based on the concern that the mechanical stimulation from the sham acupuncture needles might inadvertently elicit a mild therapeutic effect.

The present study showed that PBM did not significantly improve most of the outcomes assessed, although a slight trend toward benefit could be observed at week 8 for both the VAS score (*p* = 0.0974) and the RSFR (*p* = 0.09996). Sixty‐five percent of patients treated with PBM achieved a positive primary outcome, while only 33% of those in the sham‐PBM reached the same result. These findings were more favorable than those reported in previous studies. Zhou et al. (2022) reported an improvement on a numeric analogic scale of 28.33% of participants in the intervention group and 31.66% in the placebo group who met the primary outcome criterion (Zhou et al. 2022). Similarly, List et al. (1998) observed that traditional acupuncture significantly reduces subjective mouth dryness, which is consistent with the trend observed in the present study (List et al. 1998).

For our secondary outcome, 21.43% of patients in the PBM group showed improvement in RSFR, compared to no improvement in the sham‐PBM group at week 8. This finding is consistent with a previous study by Cafaro et al. (2015), which also used laser acupuncture to treat patients with SjD. Their results demonstrated that PBM increased saliva production compared to the placebo group (Cafaro et al. 2015). For other outcomes assessed, minimal or no significant differences were observed between the groups at both week 8 and week 12. PBM applied to acupuncture points might yield better results with continued treatment, potentially reducing the decline of clinical benefit after treatment discontinuation. Nevertheless, this would require recurrent clinical visits, resulting in additional costs for both the patients and the healthcare system.

Although some of the outcomes assessed in this study were only marginally significant, it is noteworthy that in our pilot study, in which PBM was applied to healthy individuals, 65% of participants showed an immediate improvement in RSFR after acupoint stimulation. These findings may encourage further research to evaluate the effectiveness of PBM in other xerostomia‐related conditions, considering that SjD leads to glandular destruction, which may negatively affect the response to any therapy aimed at improving salivary flow rate (Cartee et al. 2015; Mavragani and Moutsopoulos 2014; Thorne and Sutcliffe 2017).

This study has some limitations. First, the inclusion of patients with severely compromised salivary gland function may have limited the effectiveness of PBM in this subset of patients; however, this still reflects the real‐world clinical profile of individuals with SjD. Furthermore, the relatively small sample size may have reduced the statistical power to detect significant differences between groups. Higher‐than‐expected participant dropout rates may also have influenced the study results. This could be partially attributed to a lack of familiarity with TCM principles among Western populations, potentially leading to reluctance in fully engaging with the study protocol. In addition, because the trial was conducted without external funding, participants were required to cover travel and ancillary expenses. Such logistical and financial barriers are well‐recognized challenges in clinical research performed in real‐world, resource‐limited settings. Importantly, this attrition occurred similarly across groups, reducing the likelihood of systematic bias.

In conclusion, PBM demonstrated a marginal trend toward benefit at week 8 for both the VAS score and the RSFR; no significant improvements in symptoms were observed 2 months after the last treatment session. These findings suggest that PBM may have a short‐term impact on patient‐perceived disease activity and may transiently stimulate salivary gland function. Further studies with larger sample sizes are warranted to confirm these preliminary findings and to explore potential long‐term effects of PBM in Sjögren's disease. If confirmed, PBM applied to acupuncture points could represent a novel, safe, and well‐tolerated therapeutic option for patients with SjD, in contrast to current pharmacologic treatments that are often associated with adverse events.

## Author Contributions

**Matheus Ferreira Linares:** conceptualization, investigation, data curation, writing – original draft, writing – review and editing, validation, formal analysis, methodology. **Tainá Duarte dos Santos França:** writing – review and editing, writing – original draft, data curation, investigation. **Maria Clara Barros Madureira Ferreira:** investigation, writing – original draft, writing – review and editing, data curation. **Michele Gomes do Nascimento:** conceptualization, methodology, validation, formal analysis, supervision, project administration, writing – review and editing, writing – original draft. **Angela Luzia Branco Pinto Duarte:** writing – review and editing, writing – original draft, supervision, project administration. **Luiz Alcino Gueiros:** writing – original draft, writing – review and editing, supervision, methodology, conceptualization, visualization.

## Funding

This work was supported by Coordenação de Aperfeiçoamento de Pessoal de Nível Superior—CAPES [funding code 001].

## Ethics Statement

This study received ethical approval from the Research Ethics Committee of Hospital das Clínicas—UFPE (7.126.862). The study adhered to the principles of the Declaration of Helsinki. Written informed consent was obtained from all participants.

## Conflicts of Interest

The authors declare no conflicts of interest.

## Data Availability

The data that support the findings of this study are available from the corresponding author upon reasonable request.

## References

[odi70241-bib-0001] Amaral, J. P. d. A. R. , D. N. d. S. Marques , W. M. Thomson , A. R. R. Vinagre , and A. D. S. P. da Mata . 2018. “Validity and Reliability of a Portuguese Version of the Summated Xerostomia Inventory‐5.” Gerodontology 35, no. 1: 33–37. 10.1111/ger.12313. 29193291

[odi70241-bib-0002] Assy, Z. , and H. S. Brand . 2018. “A Systematic Review of the Effects of Acupuncture on Xerostomia and Hyposalivation.” BMC Complementary and Alternative Medicine 18, no. 1: 57. 10.1186/s12906-018-2124-x. PMC5811978 29439690

[odi70241-bib-0003] Baer, A. N. , and B. Walitt . 2018. “Update on Sjögren Syndrome and Other Causes of Sicca in Older Adults.” Rheumatic Disease Clinics of North America 44: 419–436. 10.1016/j.rdc.2018.03.002. PMC6245643 30001784

[odi70241-bib-0004] Baldini, C. , G. Fulvio , G. La Rocca , and F. Ferro . 2024. “Update on the Pathophysiology and Treatment of Primary Sjögren Syndrome.” Nature Reviews Rheumatology 20: 473–491. 10.1038/s41584-024-01135-3. 38982205

[odi70241-bib-0005] Biswas, R. , J. C. Ahn , J. H. Moon , et al. 2018. “Low‐Level Laser Therapy With 850 Nm Recovers Salivary Function via Membrane Redistribution of Aquaporin 5 by Reducing Intracellular ca 2+ Overload and ER Stress During Hyperglycemia.” Biochimica et Biophysica Acta ‐ General Subjects 1862, no. 8: 1770–1780. 10.1016/j.bbagen.2018.05.008. 29751100

[odi70241-bib-0006] Bowman, S. J. , R. Fox , T. Dörner , et al. 2022. “Safety and Efficacy of Subcutaneous Ianalumab (VAY736) in Patients With Primary Sjögren's Syndrome: A Randomised, Double‐Blind, Placebo‐Controlled, Phase 2b Dose‐Finding Trial.” Lancet 399, no. 10320: 161–171. 10.1016/S0140-6736(21)02251-0. 34861168

[odi70241-bib-0007] Brito‐Zerón, P. , C. Baldini , H. Bootsma , et al. 2016. “Sjögren Syndrome.” Nature Reviews Disease Primers 2: 16047. 10.1038/nrdp.2016.47. 27383445

[odi70241-bib-0008] Cafaro, A. , P. G. Arduino , A. Gambino , E. Romagnoli , and R. Broccoletti . 2015. “Effect of Laser Acupuncture on Salivary Flow Rate in Patients With Sjögren's Syndrome.” Lasers in Medical Science 30: 1805–1809. 10.1007/s10103-014-1590-8. 24820476

[odi70241-bib-0009] Cartee, D. L. , S. Maker , D. Dalonges , and M. C. Manski . 2015. “SJ ‐ cartee.” Journal of Dental Hygiene 89, no. 6: 365–371. 26684993

[odi70241-bib-0010] Choudhary, R. , S. S. Reddy , R. Nagaraju , et al. 2023. “Effectiveness of Pharmacological Interventions for Sjogren Syndrome ‐ A Systematic Review.” Journal of Clinical and Experimental Dentistry 15, no. 1: 51–63. 10.4317/jced.59891. PMC9899366 36755678

[odi70241-bib-0011] da Mata, A. , D. N. da Silva Marques , F. Freitas , et al. 2012. “Translation, Validation, and Construct Reliability of a Portuguese Version of the Xerostomia Inventory.” Oral Diseases 18, no. 3: 293–298. 10.1111/j.1601-0825.2011.01879.x. 22151408

[odi70241-bib-0012] Dabić, D. T. , S. Jurišić , V. V. Boras , D. Gabrić , I. Bago , and D. V. Vrdoljak . 2016. “The Effectiveness of Low‐Level Laser Therapy in Patients With Drug‐Induced Hyposalivation: A Pilot Study.” Photomedicine and Laser Surgery 34, no. 9: 389–393. 10.1089/pho.2016.4109. 27415181

[odi70241-bib-0013] Dawes, C. 1987. “Physiological Factors Affecting Salivary Flow Rate, Oral Sugar Clearance, and the Sensation of Dry Mouth in Man.” Journal of Dental Research 66: 648–653. 10.1177/00220345870660S107 3476629

[odi70241-bib-0014] Fidelix, T. , A. Czapkowski , S. Azjen , A. Andriolo , P. H. Neto , and V. Trevisani . 2018. “Low‐Level Laser Therapy for Xerostomia in Primary Sjögren's Syndrome: A Randomized Trial.” Clinical Rheumatology 37, no. 3: 729–736. 10.1007/s10067-017-3898-9. 29119483

[odi70241-bib-0015] Fisher, B. A. , X. Mariette , A. Papas , et al. 2024. “Safety and Efficacy of Subcutaneous Iscalimab (CFZ533) in Two Distinct Populations of Patients With Sjögren's Disease (TWINSS): Week 24 Results of a Randomised, Double‐Blind, Placebo‐Controlled, Phase 2b Dose‐Ranging Study.” Lancet 404, no. 10452: 540–553. 10.1016/S0140-6736(24)01211-X. 39096929

[odi70241-bib-0016] Furness, S. , G. Bryan , R. McMillan , and H. V. Worthington . 2013. “Interventions for the Management of Dry Mouth: Nonpharmacological Interventions.” Cochrane Database of Systematic Reviews 2013: CD009603. 10.1002/14651858.CD009603.pub2. 23996155

[odi70241-bib-0017] Gomes‐Silva, J. M. , C. P. Torres , L. R. Teixeira , et al. 2025. “Prospective Sham‐Controlled Trial: Acupuncture for Symptom‐Relieving in Patients With Sjögren's Disease.” Clinical Rheumatology 44, no. 5: 1971–1982. 10.1007/s10067-025-07410-2. 40178679

[odi70241-bib-0018] Gueiros, L. A. , K. France , R. Posey , et al. 2019. “World Workshop on Oral Medicine VII: Immunobiologics for Salivary Gland Disease in Sjögren's Syndrome: A Systematic Review.” Oral Diseases 25, no. 1: 102–110. 10.1111/odi.13062. PMC6544171 31140693

[odi70241-bib-0019] Hackett, K. L. , K. H. O. Deane , V. Strassheim , et al. 2015. “A Systematic Review of Non‐Pharmacological Interventions for Primary Sjögren's Syndrome.” Rheumatology 54, no. 11: 2025–2032. 10.1093/rheumatology/kev227. PMC4603277 26135587

[odi70241-bib-0020] Jiang, Q. , H. Zhang , R. Pang , J. Chen , Z. Liu , and X. Zhou . 2017. “Acupuncture for Primary Sjögren Syndrome (pSS) on Symptomatic Improvements: Study Protocol for a Randomized Controlled Trial.” BMC Complementary and Alternative Medicine 17, no. 1: 61. 10.1186/s12906-017-1559-9. PMC5248458 28103850

[odi70241-bib-0021] Kalhori, K. A. M. , F. Vahdatinia , M. R. Jamalpour , et al. 2019. “Photobiomodulation in Oral Medicine.” Photobiomodulation, Photomedicine, and Laser Surgery 37: 837–861. 10.1089/photob.2019.4706. 31873066

[odi70241-bib-0022] List, T. , T. Lundeberg , I. Lundström , F. Lindström , and N. Ravald . 1998. “The Effect of Acupuncture in the Treatment of Patients With Primary Sjögren's Syndrome: A Controlled Study.” Acta Odontologica Scandinavica 56, no. 2: 95–99. 10.1080/00016359850136058. 9669460

[odi70241-bib-0023] Liu, H. , X. Wang , W. Liu , G. He , X. Liang , and Y. Bian . 2023. “Effectiveness and Safety of Traditional Chinese Medicine in Treatment of Primary Sjögren's Syndrome Patients: A Meta‐Analysis.” Combinatorial Chemistry & High Throughput Screening 26, no. 14: 2554–2571. 10.2174/1386207326666230322092252. 36959129

[odi70241-bib-0024] Lončar, B. , M. Mravak Stipetić , M. Baričević , and D. Risović . 2011. “The Effect of Low‐Level Laser Therapy on Salivary Glands in Patients With Xerostomia.” Photomedicine and Laser Surgery 29, no. 3: 171–175. 10.1089/pho.2010.2792. 21054200

[odi70241-bib-0025] Ma, Y. , K. Zhou , J. Fan , and S. Sun . 2016. “Traditional Chinese Medicine: Potential Approaches From Modern Dynamical Complexity Theories.” Frontiers of Medicine 10, no. 1: 28–32. 10.1007/s11684-016-0434-2. 26809465

[odi70241-bib-0026] Mavragani, C. P. , and H. M. Moutsopoulos . 2014. “Sjögren Syndrome.” Canadian Medical Association Journal 186: E579–E586. 10.1503/cmaj.122037. PMC4203623 24566651

[odi70241-bib-0027] Mercadante, V. , R. Ní Ríordáin , S. Porter , and S. Fedele . 2016. “Determining the Minimal Clinically Important Improvement in Radiotherapy‐Induced Xerostomia.” Oral Diseases 22, no. S2: 18. 10.1111/odi.12559.

[odi70241-bib-0028] Mercadante, V. , B. J. Siri , D. K. Smith , et al. 2021. “Salivary Gland Hypofunction and/or Xerostomia Induced by Nonsurgical Cancer Therapies: ISOO/MASCC/ASCO Guideline.” Journal of Clinical Oncology 39: 18. www.asco.org/supportive‐care‐guidelines. 10.1200/JCO.21.01208 34283635

[odi70241-bib-0029] Palma, L. F. , F. A. S. Gonnelli , M. Marcucci , et al. 2017. “Impact of Low‐Level Laser Therapy on Hyposalivation, Salivary pH, and Quality of Life in Head and Neck Cancer Patients Post‐Radiotherapy.” Lasers in Medical Science 32, no. 4: 827–832. 10.1007/s10103-017-2180-3. 28258315

[odi70241-bib-0030] Pandeshwar, P. , M. D. Roa , R. Das , S. P. Shastry , R. Kaul , and M. B. Srinivasreddy . 2016. “Photobiomodulation in Oral Medicine: A Review.” Journal of Investigative and Clinical Dentistry 7: 114–126. 10.1111/jicd.12148. 25720555

[odi70241-bib-0031] Petruzzi, M. , F. della Vella , N. Squicciarini , et al. 2023. “Diagnostic Delay in Autoimmune Oral Diseases.” Oral Diseases 29: 2614–2623. 10.1111/odi.14480. 36565434

[odi70241-bib-0032] Plemons, J. M. , I. Al‐Hashimi , and C. L. Marek . 2014. “Managing Xerostomia and Salivary Gland Hypofunction: Executive Summary of a Report From the American Dental Association Council on Scientific Affairs.” Journal of the American Dental Association 145, no. 8: 867–873. 10.14219/jada.2014.44. 25082939

[odi70241-bib-0033] Ramos‐Casals, M. , P. Brito‐Zerón , A. Sisó‐Almirall , and X. Bosch . 2012. “Primary Sjögren Syndrome.” BMJ 345, no. 7872: e3821. 10.1136/bmj.e3821. 22700787

[odi70241-bib-0034] Seror, R. , P. Ravaud , X. Mariette , et al. 2011. “EULAR Sjögren's Syndrome Patient Reported Index (ESSPRI): Development of a Consensus Patient Index for Primary Sjögren's Syndrome.” Annals of the Rheumatic Diseases 70, no. 6: 968–972. 10.1136/ard.2010.143743. 21345815

[odi70241-bib-0035] Seror, R. , E. Theander , J. G. Brun , et al. 2015. “Validation of EULAR Primary Sjögren's Syndrome Disease Activity (ESSDAI) and Patient Indexes (ESSPRI).” Annals of the Rheumatic Diseases 74: 859–866. 10.1136/annrheumdis-2013-204615. 24442883

[odi70241-bib-0036] Shiboski, C. H. , S. C. Shiboski , R. Seror , et al. 2017. “2016 American College of Rheumatology/European League Against Rheumatism Classification Criteria for Primary Sjögren's Syndrome: A Consensus and Data‐Driven Methodology Involving Three International Patient Cohorts.” Annals of the Rheumatic Diseases 76, no. 1: 9–16. 10.1136/annrheumdis-2016-210571. 27789466

[odi70241-bib-0037] Sousa, A. S. , J. F. Silva , V. C. S. Pavesi , et al. 2020. “Photobiomodulation and Salivary Glands: A Systematic Review.” Lasers in Medical Science 35: 777–788. 10.1007/s10103-019-02914-1. 31768691

[odi70241-bib-0038] Tang, J.‐L. , B.‐Y. Liu , and K.‐W. Ma . 2008. “Traditional Chinese Medicine.” Lancet 372, no. 9654: 1938–1940. 10.1016/S0140-6736(08)61354-9. 18930523

[odi70241-bib-0039] Thomson, W. M. 2007. “Measuring Change in Dry‐Mouth Symptoms Over Time Using the Xerostomia Inventory.” Gerodontology 24, no. 1: 30–35. 10.1111/j.1741-2358.2007.00137.x. 17302928

[odi70241-bib-0040] Thorlacius, G. E. , A. Björk , and M. Wahren‐Herlenius . 2023. “Genetics and Epigenetics of Primary Sjögren Syndrome: Implications for Future Therapies.” Nature Reviews Rheumatology 19: 288–306. 10.1038/s41584-023-00932-6. PMC10010657 36914790

[odi70241-bib-0041] Thorne, I. , and N. Sutcliffe . 2017. “Thorne 2017.” British Journal of Hospital Medicine 78, no. 8: 438–442. 10.12968/hmed.2017.78.8.438. 28783408

[odi70241-bib-0042] Wei, W. , S. S. Ahmad , S. Chi , Y. Xie , M. A. Kamal , and J. Li . 2018. “From Molecular Mechanism to the Etiology of Sjogren Syndrome.” Current Pharmaceutical Design 24, no. 35: 4177–4185. 10.2174/1381612824666181016154033. 30332943

[odi70241-bib-0043] Wu, D. , X. Lan , G. Litscher , et al. 2024. “Laser Acupuncture and Photobiomodulation Therapy in Bell's Palsy With a Duration of Greater Than 8 Weeks: A Randomized Controlled Trial.” Lasers in Medical Science 39, no. 1: 29. 10.1007/s10103-023-03970-4. PMC10787006 38216803

[odi70241-bib-0044] Wu, J. , and D. Li . 2024. “Pilocarpine Treatment of Sjögren's Syndrome Curative Effect of Meta‐Analysis of Randomised Controlled Trials Pilocarpine Treatment of Sjögren's Syndrome.” Clinical and Experimental Rheumatology 42. 10.55563/clinexprheumatol/lkk9qq. 39699871

[odi70241-bib-0045] Wu, S. Y. , C. H. Lin , N. J. Chang , et al. 2020. “Combined Effect of Laser Acupuncture and Electroacupuncture in Knee Osteoarthritis Patients: A Protocol for a Randomized Controlled Trial.” Medicine 99, no. 12: 2490–2498. 10.1097/MD.0000000000019541. PMC7220484 32195960

[odi70241-bib-0046] Zecha, J. A. E. M. , J. E. Raber‐Durlacher , R. G. Nair , et al. 2016a. “Low‐Level Laser Therapy/Photobiomodulation in the Management of Side Effects of Chemoradiation Therapy in Head and Neck Cancer: Part 2: Proposed Applications and Treatment Protocols.” Supportive Care in Cancer 24: 2793–2805. 10.1007/s00520-016-3153-y. PMC4846551 26984249

[odi70241-bib-0047] Zecha, J. A. E. M. , J. E. Raber‐Durlacher , R. G. Nair , et al. 2016b. “Low Level Laser Therapy/Photobiomodulation in the Management of Side Effects of Chemoradiation Therapy in Head and Neck Cancer: Part 1: Mechanisms of Action, Dosimetric, and Safety Considerations.” Supportive Care in Cancer 24: 2781–2792. 10.1007/s00520-016-3152-z. PMC4846477 26984240

[odi70241-bib-0048] Zhao, Z. Q. 2008. “Neural Mechanism Underlying Acupuncture Analgesia.” Progress in Neurobiology 85: 355–375. 10.1016/j.pneurobio.2008.05.004. 18582529

[odi70241-bib-0049] Zhou, X. , H. Xu , J. Chen , et al. 2022. “Efficacy and Safety of Acupuncture on Symptomatic Improvement in Primary Sjögren's Syndrome: A Randomized Controlled Trial.” Frontiers in Medicine 9: 878218. 10.3389/fmed.2022.878218. PMC9121854 35602489

